# Viscoelastic Hemostatic Assays and Platelet Function Testing in Patients with Atherosclerotic Vascular Diseases

**DOI:** 10.3390/diagnostics11010143

**Published:** 2021-01-19

**Authors:** Matej Samoš, Ingrid Škorňová, Tomáš Bolek, Lucia Stančiaková, Barbora Korpallová, Peter Galajda, Ján Staško, Peter Kubisz, Marián Mokáň

**Affiliations:** 1 Department of Internal Medicine I, Jessenius Faculty of Medicine in Martin, Comenius University in Bratislava, 036 59 Martin, Slovakia; ato.bolek@gmail.com (T.B.); barbora.korpallova@gmail.com (B.K.); peter.galajda@uniba.sk (P.G.); mokanmarian@gmail.com (M.M.); 2 National Centre of Hemostasis and Thrombosis, Department of Hematology and Blood Transfusion, Jessenius Faculty of Medicine in Martin, Comenius University in Bratislava, 036 59 Martin, Slovakia; inkaskornova@gmail.com (I.Š.); lstanciakova@gmail.com (L.S.); jan.stasko@uniba.sk (J.S.); peter.kubisz@uniba.sk (P.K.)

**Keywords:** viscoelastic hemostatic assays, thromboelastometry, thromboelastography, platelet function testing, atherosclerotic vascular disease

## Abstract

Platelets play crucial role in acute vascular atherosclerotic diseases, including myocardial infarction and stroke. Additionally, platelet aggregation is a key target of antiplatelet agents, forming the keystone of pharmacotherapy of various atherosclerotic cardiovascular diseases. Thromboelastography and thromboelastometry, representing currently available viscoelastic hemostatic assays (VHA), are designed as whole blood, real-time analyzers of clot formation and clot resolution. These assays could, in theory, overcome some limitations of currently available platelet function testing assays. This article reviews the current experience with the use of VHA for platelet function testing and for monitoring of the response to antiplatelet therapy.

## 1. Introduction

Activation and aggregation of platelets plays an important role in acute vascular atherosclerotic diseases, including myocardial infarction and stroke. Additionally, platelet aggregation is a key target of antiplatelet agents, such as acetylsalicylic acid (ASA, aspirin), P2Y12 adenosinediphosphate (ADP) receptor blockers (ADPRB) and glycoprotein IIb/IIIa blockers. Thus, these agents are often used to treat or to prevent atherosclerotic vascular disease; nevertheless, the insufficient response to these agents (high on-treatment platelet reactivity (HPR), or antiplatelet therapy resistance) has been repeatedly connected with higher risk of vascular events, and increased mortality. This raises the question about the need for platelet function testing to identify individuals with HPR or impaired responses to antiplatelet drugs. Although several methods, such as light transmission or impedance aggregometry, vasodilator-stimulated phosphoprotein (VASP) phosphorylation assessment, the Platelet Function Analyzer (PFA)-100^®^ assay or Verify Now^®^ assay have been introduced for platelet function/antiplatelet therapy response testing [1,2,3], these methods have their disadvantages.

Viscoelastic hemostatic assays (VHA), available as thromboelastography and thromboelastometry, are designed as whole-blood, real-time analyzers of clot formation and lysis [4,5]. These assays are, in theory, capable of overcoming some limitations of currently available platelet function tests, such as longer turnaround times, the need for special equipment and skilled staff, and an inability to test the platelet function in point-of-care settings. This article reviews current experience with the use of VHA for platelet function testing (PFT) and for monitoring of the response to antiplatelet drugs in patients with atherosclerotic vascular diseases.

## 2. Viscoelastic Hemostatic Assays and Platelet Function Testing

### 2.1. Platelet Dysfunction and Adverse Vascular Events

As already mentioned, platelet dysfunction (abnormal platelet activation and aggregation) plays important role in cardiac adverse events [6]. HPR predicts future ischemia in patients undergoing percutaneous coronary interventions (PCI) for acute coronary syndromes (ACS) [7,8,9], as well as in those after coronary stenting for stable coronary artery disease (SCAD) [10,11,12]. Moreover, HPR seems to be related to vascular dysfunction in patients with SCAD on antiplatelet agents [13], and platelet activation after PCI could independently predict worse clinical outcome and may be helpful in risk stratification [14]. In another study, high mean platelet volume—a simple indicator of platelet size that correlates with platelet activation and aggregability—was associated with a significantly increased incidence of long-term adverse events [15]. In addition, platelet dysfunction and abnormal (high) platelet activation or abnormal platelet aggregation is independently associated with the risk of ischemic stroke [16,17,18], lower limb ischemia [19], diabetic vascular disease [18,20] and vasculogenic erectile dysfunction [21]. Summarizing, platelet dysfunction with abnormal platelet activation and aggregation is connected with various cardiovascular ischemic diseases. Therefore, unsurprisingly, antiplatelet therapy forms currently the keystone of pharmacology of these diseases.

Going further, inadequate therapeutic response—or so called HPR on P2Y12 ADPRB [8,9,10,11,12,14,22,23,24,25], and aspirin [25,26,27] (with a lower level of evidence)—is associated with unfavorable clinical course, and predicts independently future thrombotic adverse events in those undergoing coronary stent implantation (both for ACS and SCAD). For example, in a sub-analysis of the randomized GRAVITAS (Gauging Responsiveness with a Verify Now P2Y12 assay: Impact on Thrombosis and Safety) study [24], HPR (defined as >208 P2Y12 reaction units when examined with a point-of-care Verify Now^®^ assay at 12 to 24 h after PCI) was connected with a higher risk of adverse ischemia at 60 days and at 6 months (at 60 days: hazard ratio [HR]: 0.18; 95% confidence interval [CI]: 0.04–0.79; *p* = 0.02; at 6 months: HR: 0.43; 95% CI: 0.23–0.82; *p* = 0.01). Additionally, HPR remains independently associated with adverse vascular events (including stent thrombosis) also in individuals treated with novel P2Y12 ADP receptor blockers with stronger platelet inhibition, such as prasugrel and ticagrelor [28,29,30]. Summarizing the data, HPR despite oral antiplatelet agents has been consistently connected with the risk of adverse ischemia; thus identifying individuals with insufficient antiplatelet therapy response might help in preventing these possibly fatal events. Several platelet function tests have been introduced for this purpose.

### 2.2. Platelet Function Testing in Cardiovascular Medicine

As already discussed, PFT might be used to search for patients with HPR and to predict platelet response to ASA and P2Y12 ADPRB therapy. In addition, several previously published papers pointed to the fact that this testing might be useful for the identification and management of platelet-related bleeding during cardiac [31,32,33,34,35] and non-cardiac surgery [36,37], or to identify patients with inherited platelet disorders [38,39]. Several platelet function tests have been established for PFT in cardiovascular diseases. These include light transmission and multiple electrode aggregometry [40], the Verify Now^®^ assay [41,42], VASP phosphorylation (VASP-P) flow cytometric assessment [43], and PFA-100^®^ analysis [40].

Light-transmission (optical) aggregometry (LTA), a turbidometry-based assay, still represents a “gold standard” in PFT [40]. The assay assesses platelet aggregation in platelet-rich plasma (PRP), the aggregation is induced by non-specific (collagen, epinephrine) or drug-specific (arachidonic acid, ADP) inducers (platelet agonists). The assay detects differences (with a photometer) in light transmission after a selected inducer is added to PRP. Measurement of aggregation is displayed as aggregation curve which presents the intensity of changes in the light transmission of examined PRP. The maximal extent of platelet aggregation is then expressed in percentages (0% represents no aggregation, 100% represents maximal possible platelet aggregation). Samples can be tested with a large scale (and at different concentrations) of inducers, which allows different pathways of platelet activation to be tested, or treatment response to different antiplatelet agents to be tested. The major disadvantages of the assay include a need for special laboratory equipment and specially skilled laboratory staff to perform the analysis, the fact that the analysis itself is time-consuming, the pre-analytical demands of the assay (PRP needs to be prepared and analyzed within one hour of blood sampling), and the non-specificity of the method.

Multiple electrode (impedance) aggregometry [40] assesses changes in electrical impedance between multiple electrodes after inducing the aggregation by a platelet inducer. The method is similar to LTA except that it uses a whole blood sample, and thus there is no need to prepare PRP. The assay can be used in point-of-care (POC) settings, and contains five channels for simultaneous analysis of different samples or simultaneous analysis of one sample with different inducers. The assay is more cost-demanding compared to LTA, and the platelet response to the inducer is non-specific.

The Verify Now^®^ (Accumetrics, San Diego, CA, USA) system is a POC assay [42] based on a modified aggregometry with a use of drug-specific inducers (arachidonic acid, ADP) and thrombin receptor-activating peptide (TRAP). The assay assesses the agglutination of fibrinogen-coated beads by platelets stimulated by the inducer in whole blood sample with citrate. The Verify Now^®^ P2Y12 assay detects the extent of the P2Y12 blockage by a P2Y12 ADPRB. The system has two assay channels. P2Y12 ADPRB inhibits aggregation in the ADP-containing channel but not in the second channel with TRAP. Aggregation in both channels is assessed as the change (increase) in light transmission and is reported in PRU (platelet response units). Likewise, the Verify Now^®^ Aspirin assay [41] contains fibrinogen-coated beads and arachidonic acid (specific inducer), platelet activation is induced with specific inducer, and activated platelets then bind to the fibrinogen-coated beads causing agglutination. The degree of light transmission is proportional to aggregation; ASA inhibits this agglutination. The assay is quick, and user friendly; nevertheless, it is less sensitive to the platelet ADP signaling pathway compared with VASP-P analysis [44].

VASP-P assessment [43] accurately and specifically detects platelet response on P2Y12 ADP receptor blockers. The test is performed ex vivo on a flow cytometer. A sample of citrated blood is incubated with prostaglandin E1 and with prostaglandin E1 plus ADP. After cellular permeabilization, VASP-P is labeled by immunofluorescence using a specific monoclonal antibody. Dual color flow cytometrythen allows final calculation of the “platelet reactivity index” (PRI) using corrected VASP fluorescence intensities in the presence of prostaglandin E1 alone (resting) or prostaglandin E1 plus ADP (activated platelets). The major advantage of the assay is its specificity for the ADP signaling pathway. However, examination requires a laboratory equipped with a flow cytometer and skilled laboratory staff, and is cost-demanding. These disadvantages limit its use in daily clinical practice.

PFA-100^®^ (Siemens, Munich, Germany) assesses the cessation of high-shear blood flow by the platelet plug [40]. A quick and simple POC assay needs a low volume of sample and no sample preparation. In this method, whole blood with citrate flows through a capillary at a high shear rate within the test cartridge that ends in a membrane filled with a platelet agonist (inducer). The time until clot build-up occludes the aperture (closure time) correlates with platelet aggregation rate. The examination itself can be performed with cartridges containing different inducers—collagen, ADP, prostaglandin E1, epinephrine—which can be used, similar to LTA, to test different pathways of platelet activation. The disadvantages of the method are that it is dependent on hematocrit and von Willebrand factor levels, the non-specificity of platelet activation during the test, and that it requires pipetting.

Considering the aforementioned disadvantages of already available platelet function tests, the key question of this article is whether VHA assay-based platelet function testing might provide additional benefits in clinical management of platelet dysfunction or antiplatelet drugs in patients with atherosclerotic vascular diseases (compared to already available platelet function testing).

### 2.3. Thromboelastography and Thromboelastometry: Assays Principle, Advantages and Disadvantages

**Figure 1:**
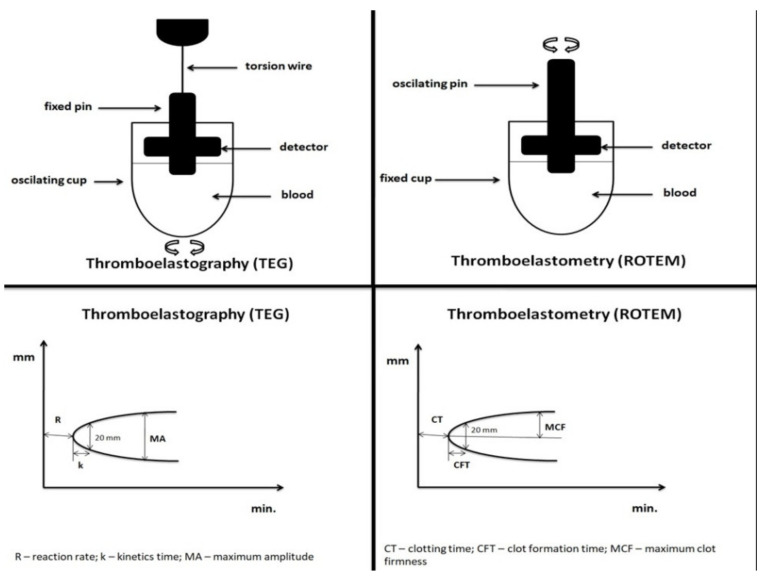
Thromboelastography (TEG) and rotational thromboelastometry (ROTEM).

Both thromboelastography (TEG^®^, Haemoscope, Haemonetics, Niles, IL, USA) and rotational thromboelastometry (ROTEM^®^, Instrumentation Laboratory, Bedford, MA, USA) are designed as POC VHA that use a whole blood sample, which is examined in real time, with slight differences in operating mechanisms. TEG^®^ (Table 1) and ROTEM^®^ (Table 2) assess clot formation, clot lysis and clot strength by assessing and displaying the amount of a continuously applied rotational force that is transmitted to an electromechanical transduction system by the forming clot (Figure 1). In the thromboelastography, a rotating cylindrical cup (with a 340 µL whole blood sample) oscillates through 4°45′ every 5 s while a pin on a torsion wire is fixed in the blood sample. As the strength of the forming clot increases, more rotation is transmitted to the wire and is recognized by a transducer. In the thromboelastometry system, a cylindrical cup (with a 340 µL of whole blood sample) stands fixed while a pin swingingon a ball-bearing mechanism oscillates through 4°75′ every 6 s applying a constant force. As the viscoelastic clot strength increases, the (pin) rotation is blocked and is displayed with a sensor (a charge-coupled device image sensor system) [4,45,46].

Both assays [47] graphically record kinetic changes of citrated whole blood samples during clot forming and its dissolution in the form of thromboelastogram, mapping various phases of hemostasis. The technology leans on a constantly oscillating cup/pin in a blood sample with reagents. There is no rotation blockage, if there is no clot formation. In a forming clot, there is a rotation blockage due to a link between the cup wall and pin. Thus, free rotation corresponds to an amplitude of 0 mm and no rotation corresponds to an amplitude of 100 mm. The interpreting software determines clot formation parameters in real time during the test, and these parameters are subsequently graphically interpreted in the thromboelastogram (Figure 1).

A thromboelastographydevice can analyze two samples at the same time; a thromboelastometry device can analyze four samples at the same time. While both thromboelastography and thromboelastometry provide, in general, the same data on the formation and strength of thrombi, these data are not interchangeable. This is due to different assays and coagulation inducers, as well as due to different nomenclature [46]. The most important advantages of these VHA are rapidity, complexity, and the ability to test a whole blood sample in a point-of-care setting.

Both assays have their limitations [4]. In fact, hemostasis is associated with a wide range of normal values, as there is a high variability in the components of hemostasis (platelet count, platelet activity, fibrinogene levels, glycoprotein IIb/IIIa receptor number, etc.). Therefore, in optimal settings, each patient should have baseline VHA values before the therapy or procedure to create an internal, personalized reference value for comparison. Additionally, there are difficulties with assay standardization and validation (as the method continues to diversify in activators, modifications and equipment). Thromboelastography, as well as thromboelastometry may be performed with a large scale of inducers and inhibitors which might alter the specificity of the assay. These issues have been, in part, overcome by the use of computer software analysis allowing better standardization. Further achievement in standardization has been made by the use of individual temperature control, by the use of disposable cups and pins, and the use of standard activators of coagulation (such as kaolin).

### 2.4. Platelet Function Testing with Thromboelastography

The TEG^®^ Platelet Mapping™ Assay (Haemoscope Corporation, Niles, IL, US) is a thromboelastography assay designed for PFT which relies on evaluation of clot strength to allow a quantitative measurement of platelet function. Using this assay, the maximal haemostatic activity is assessed in a kaolin activated whole blood sample with citrate. Assay performs measurements with heparin to eliminate thrombin activity [48], the contribution of the ADP or thromboxane A2 platelet receptors to the clotting is tested by the addition of arachidonic acid or ADP. The assay was evaluated in several previously published studies. First, Tantry et al. [48] evaluated the sensitivity of the assay for detection of ASA resistance (compared to arachidonic acid-induced platelet aggregation measured with LTA). In this study, enrolling 223 patients in long-term ASA therapy and six healthy individuals who received 325 mg of aspirin, the platelet aggregation assessed by LTA strongly correlated with aggregation assessed in the whole blood by the TEG^®^ Platelet Mapping™ assay (r = 0.85, *p*< 0.001). All of the patients with non-compliance (7 patients) had high platelet aggregation by both methods (TEG and LTA). Subsequently, Alström et al. [49] tested the effect of a clopidogrel bolus dose on platelet inhibition in 28 patients on aspirin therapy undergoing coronary angiography/PCI with the use of TEG^®^ Platelet Mapping™ assay and found significantly (*p* = 0.002) decreased platelet reactivity (platelet inhibition) with platelet mapping. Bochsen et al. [50,51] examined maximal platelet reactivity (MPR) and platelet reactivity response to ADP stimulation using thormboelastography with platelet mapping in healthy blood donors (43 individuals) and patients planned for coronary artery bypass graft (CABG) surgery (22 patients) and demonstrated that MPR and platelet response to ADP was higher in the CABG patients than in the healthy controls, speculating that this observation may reflect the higher risk for ischemia in the cohort of CABG patients. In another prospective observational study [52] the authors assessed platelet reactivity with TEG^®^ Platelet Mapping™ in patients presenting for orthopedic trauma surgery or acute general surgery who received clopidogrel (21 patients), aspirin (18 patients) or no antiplatelet therapy (20 patients) prior admission. In this study, TEG^®^ Platelet Mapping™ identified statistically significant inhibition of platelets due to antiplatelet therapy. Nevertheless, the authors reported an overlap in platelet receptor inhibition between the three examined groups of patients, which could, in their opinion, limit the clinical utility of the assay. Subsequently, several other studies reported that the use of TEG^®^ Platelet Mapping™ could predict postoperative blood loss among patients who underwent cardiac [53,54,55,56,57,58,59], trauma or other non-cardiac surgery [60,61,62,63,64] and received antiplatelet therapy.

Furthermore, several other studies or cases addressed the issue of monitoring the response on aspirin [65,66,67], clopidogrel [68,69,70,71,72] and ticagrelor [72,73,74] with TEG^®^ Platelet Mapping™ assay. Looking at the most recent studies, Cheng et al. [71] reported a usefulness of several TEG^®^ Platelet Mapping™ parameters (including Net platelet clot strength as the strongest predictor) for determining clopidogrel HPR in post-PCI patients. Yang et al. [72] tested the effect of clopidogrel and ticagrelor on platelet activity in patients with transient ischemic attack or minor stroke. Platelet on-treatment response was assessed with TEG^®^ Platelet Mapping™. The primary outcome of this study was the number of patients with HPR (maximum amplitude induced by adenosine diphosphate > 47 mm). The study enrolled 339 patients, 170 were randomized to ticagrelor plus ASA and 169 to clopidogrel plus ASA. The authors reported that, compared with clopidogrel, the rate of HPR in ticagrelor-treated patients was significantly lower (12.2% versus 30.0%, *p* < 0.001). Additionally, in this study, ticagrelor was superior in platelet inhibition measured by the TEG^®^ Platelet Mapping™ assay. Finally, Zhang et al. [75] reported in their analysis of 467 consecutive patients with myocardial infarction undergoing PCI that by receiver operating characteristic curve analysis, the TEG^®^ Platelet Mapping™ assay rate of ADP inhibition had the best predictive value of future hemorrhagic events. This suggests possible role of the assay in predicting bleeding (not only to predict ischemia) in patients after recent PCI.

Summarizing this issue, currently available data suggest possible clinical utility of the TEG^®^ Platelet Mapping™ assay in the settings of predicting peri-operative blood loss in patients who take antiplatelet medication and need cardiac or non-cardiac surgery, and in the prediction of treatment response (identification of antiplatelet therapy resistance) on most commonly used antiplatelet agents (aspirin, clopidogrel and ticagrelor).

### 2.5. Platelet Function Testing with Thromboelastometry

Thromboelastometry is another VHA available in routine clinical practice. This method, as mentioned, differs in the method of clot strength detection (a rotating pin in a fixed cup in thromboelastometry opposed to a fixed pin and a rotating cup in thromboelastography), reagents (with different coagulation inducers) and slightly in measured parameters. In fact, there are limited experiences in platelet function testing with thromboelastometry (compared to thromboelastography). First, the assay has been tested for the evaluation of treatment response on antiplatelet therapy. In one of the first analyses of the use of thromboelastometry for measuring the response to dual antiplatelet therapy (aspirin plus clopidogrel), we performed a pilot prospective study [76] in which samples from acute ST elevation myocardial infarction (STEMI) patients (on with dual antiplatelet therapy) were analyzed with thromboelastometry (using EXTEM^®^ and FIBTEM^®^ reagents) and compared with samples from healthy blood donors (with no antithrombotic therapy). In this study, clotting time was significantly prolonged and maximum clot firmness was significantly higher in patients compared to controls. Nevertheless, when a sub-analysis of patients with acute MI was performed, using ROTEM^®^ analysis, we were not able to discriminate HPR and normal response to antiplatelet therapy. This observation suggested that for antiplatelet therapy response testing a more specific “platelet mapping assay” for thromboelastometry would be needed. At the time of the study, there was no such assay validated. Nevertheless, Scharbert et al. [77] reported their first experiences with modified rotational thromboelastometry with platelet mapping assay prior to our study. In this study, platelet aggregability was determined from blood samples taken from 22 adult healthy individuals and patients without or with antiplatelet drugs (clopidogrel with or without aspirin) using three different assays: multiple electrode aggregometry, thromboelastography with platelet mapping assay using both inducers—ADP and arachidonic acid, and its adapted version in rotational thromboelastometry. The authors of this study reported that the specificity for detection of ADP receptor blocker-induced platelet inhibition was low in both assays; however, the differences in frequency distribution between the results obtained in thromboelastography and thromboelastometry were not statistically significant. Subsequently, Braun at al. [78] studied the antiplatelet and anticoagulant action of clopidogrel plus heparin (26 patients) versus prasugrel plus bivalirudin (25 patients) versus untreated controls (20 individuals) with multiple electrode aggregometry and rotational thromboelastometry in patients with STEMI. In this study, in rotational thromboelastometry analysis, there were no differences in reducing clot formation time between studied antiplatelet/anticoagulation agent regimens and both regimens did not affect maximum clot firmness compared to the control group. This observation confirmed our observation of low sensitivity of standard assays of rotational thromboelastometry in detecting P2Y12 receptor blocker-induced changes in platelet reactivity. Unfortunately, to date, there has been no other larger study evaluating the ability of rotational thromboelastometry with a platelet mapping assay (which was originally developed for thromboelastography) to assess the response on antiplatelet agents in patients undergoing invasive vascular procedures. Furthermore, clinical experiences with platelet function test specifically designed for thromboelastometry are very limited [79], with one prospective study [80]. This novel method is designed as a combination of standard thromboelastometry and whole blood impedance aggregometry assessed on the ROTEM platelet module (Instrumentation Laboratory, Werfen, Munich, Germany), with the use of platelet-specific reagents (containing specific platelet inducer) [79,80]. ADPTEM reagent contains ADP, ARATEM reagent contains arachidonic acid, and TRAPTEM reagent contains thrombin receptor-activating peptide. First experience with the assay was reported by Nissen et al. [80] who studied the assay on blood samples (with citrate or hirudin) collected from 121 healthy individuals and tried to establish reference intervals for platelet aggregation for each of the reagents (ADPTEM, ARATEM and TRAPTEM). The authors reported that in citrate tubes, the stability of the ROTEM platelet assays was 60–120min, while the stability in hirudin tubes was 30–60 min and established combined and gender-specific reference intervals for all three assays. Nevertheless, there are no other reports of clinical experiences with this assay and, therefore, the assay still needs appropriate validation.

Similarly, with thromboelastography, several studies tried to evaluate the use of rotational thromboelastometry for procedural bleeding risk assessment and blood replacement therapy in patients undergoing cardiac surgeries. Looking more closely at these trials, Ogawa et al. [81] observed a significant correlation between thromboelastometry test FIBTEM-amplitude at 10 min and fibrinogen levels (r = 0.87; *p* < 0.001) and between test EXTEM and INTEM-A10 (amplitude at 10 min after the initiation of clot formation) values and platelet count (r = 0.72 and 0.67; *p* < 0.001) in patients undergoing procedures with cardiopulmonary bypass. The authors suggested the ability of rotational thromboelastometry testing with FIBTEM assay to quickly detect decreasing levels of fibrinogen in patients who underwent cardiopulmonary bypass. In another study enrolling patients undergoing CABG surgery on antiplatelet agents (aspirin alone or aspirin plus clopidogrel), Tarzia et al. [82] reported an observation of comparable pre-operative classic coagulation tests and traditional ROTEM parameters in those with major bleeding and those without a bleeding event. Nevertheless, the authors reported that the area-under-the-curve in the EXTEM test was significantly lower in patients with major hemorrhage than non-bleeders. Finally, Azarfarin et al. [83] studied the relation between maximum clot firmness in rotational thromboelastometry and bleeding after CABG surgery in patients on clopidogrel and found that the need for chest tube drainage and for blood product transfusion were significantly higher in those with maximum clot firmness below 50 mm. Furthermore, in patients who experienced postoperative bleeding of 1 L of blood or more, several rotational thromboelastometry parameters including maximum clot firmness (tested with INTEM, EXTEM and HEPTEM reagents) were significantly lower than in those with postoperative bleeding < 1 L. This observation suggested that rotational thromboelastometry might be useful for the prediction of increased risk of hemorrhage after CABG in patients on clopidogrel prior to the procedure.

Concluding this issue, compared with thromboelastography, there has been no study demonstrating the ability of rotational thromboelastometry to monitor the treatment response to antiplatelet drugs or to search for patients with HPR; however, several studies (although on limited samples) suggested that it can be used for procedural management of bleeding events in the settings of cardiac surgery.

### 2.6. Clinical Experiences with Viscoelastic Hemostatic Assays (VHA) for Platelet Function Testing and Gaps in Evidence

First, looking on the utility of VHA for PFT and monitoring of the response on antiplatelet drugs, the majority of evidence is for thromboelastography, for which assay-specific platelet mapping had been developed and is commercially available. As mentioned, the evidence for thromboelastometry is still limited to several low-sample studies reporting its usefulness for prediction of platelet-related bleeding and management of blood product transfusion in cardiac surgery. Second, looking more closely at the evidence for thromboelastography, one must conclude that the evidence is coming mostly from small sample non-randomized trials and clinical cases, and that there is no larger study confirming the results. This should always be taken into consideration when VHA is selected as a test for platelet function testing. Furthermore, although thromboelastography has been generally demonstrated as effective for prediction of antiplatelet drug response and for prediction of bleeding in patients on antiplatelet agents who need surgical (cardiac or non-cardiac) procedures, there are contradictions regarding these issues. Corliss et al. [84] reported poor correlation between the results of thromboelastography with a platelet mapping assay and Verify Now^®^ analysis of platelet reactivity in patients who underwent cranial endovascular stenting procedures. In this retrospective single centre study, Verify Now^®^ analysis accurately predicted thrombotic and hemorrhagic complications, while the maximal amplitude of thromboelastography ADP platelet mapping assay was not able to accurately predict these complications. In another study, Lam et al. [85] reported that thromboelastography with platelet mapping was, in their analysis of 141 patients with spontaneous intra-cerebral bleeding, unable to measure platelet inhibition (in those who were on antiplatelet agents) and was not a reliable method to guide platelet transfusion. Similar to this observation, Daley et al. [86] retrospectively analyzed a sample of 129 patients with acute trauma reported that there was no significant difference in thromboelastographic parameters of platelet function, including rate of arachidonic acid or ADP inhibition between patients on antiplatelet agents prior to injury and patients who did not take antiplatelet agents prior acute traumatic event. Therefore, thrombolastography alone should probably not be used to estimate the presence of antiplatelet drugs and, knowing the information for a patient taking an antiplatelet drug is still important. The question is what causes these contradictions. In fact, up to date, there is no satisfactory answer to this question. The most probable explanation is that the method has an apparent lack of specificity, as the initiation and propagation of coagulation process in VHA, including that with a platelet mapping assay, is unspecific and could be affected by various other confounding factors (such as platelet count, fibrinogen levels, liver dysfunction, tissue factor levels, etc.).

Second, there is an issue of correlation between TEG^®^ Platelet Mapping™ Assay with other platelet function assays. In some respects, TEG platelet mapping could be inferior to VASP-P flow cytometry, considering the assay principles [43]. In fact, there is only one study directly evaluating the TEG Platelet Mapping Assay together with VASP-P flow cytometry in a sample of healthy volunteers who received clopidogrel therapy and three different proton pump inhibitors. In this study, Lee et al. [87] reported that both VASP-P and TEG platelet mapping showed the same general qualitative trend, but TEG^®^ Platelet Mapping™ Assay detected a significant fluctuation of platelet aggregation in response to different proton pump inhibitors/clopidogrel interaction. As there is no other study comparing these two methods, this issue remains open for future resolution. Furthermore, when comparing TEG platelet mapping with Verify Now^®^ and impedance aggregometry (Multiplate^®^), TEG platelet mapping often comes third among these assays. This could be due to the fact that clinical experience with Verify Now^®^ and Multiplate^®^ is higher compared to TEG platelet mapping. However, when these three assays were compared with one another in 64 adult trauma patients (25 patients were taking antiplatelet drugs) in an observational study performed by Connelly et al. [88], there were no significant differences between assays and TEG^®^ Platelet Mapping™ Assay, Verify Now^®^ and Multiplate^®^ correlated well and were able to identify aspirin-induced platelet dysfunction. On the other hand, in another study evaluating aspirin-induced platelet dysfunction by LTA, Verify Now^®^, Multiplate impedance aggregometry and TEG^®^ Platelet Mapping™ Assay and clopidogrel-induced platelet dysfunction by the same methods and by VASP-P flow cytometry [89], the TEG^®^ Platelet Mapping™ Assay was least suited to monitor antiplatelet agents-induced platelet dysfunction. Thus further studies are needed to raise final conclusions.

Third, all the platelet function tests based on platelet in vitro induction, including VHA with platelet mapping, measure platelet aggregation (as this is the key target of antiplatelet agents); they do not measure platelet adhesion. Therefore, it is necessary to use flow chamber techniques for the assessment of platelet adhesion [90]. Although the measurement of platelet adhesion is not generally needed in clinical practice, there are several clinical situations, in which impaired platelet adhesion is responsible for platelet dysfunction or platelet-related adverse events [91,92].

Fourth, it is not entirely clear what anticoagulation (citrated or hirudin or other) should be used for VHA platelet mapping. In a previous study the authors using blood samples from healthy volunteers compared LTA aggregation with different inducers (ADP, arachidonic acid, collagen, epinephrine and ristocetin) in samples collected to tubes with 3.2% trisodium citrate versus to tubes with recombinant hirudin (>15 µg/mL) [93]. The authors of this study reported that among all the agonists, hirudin-anticoagulated platelets had significantly weaker aggregation responses, possibly preferring citrated tubes for LTA platelet function testing. This should probably be applied also for VHA platelet mapping [48,80]; nevertheless, there is no study directly examining this issue in VHA platelet mapping, and therefore, there is no study for confirming this recommendation.

Concluding these issues, further research intothe use of VHA in platelet function testing is still warranted.

## 3. Conclusions

Currently available data suggest possible clinical utility of thromboelastography with platelet mapping assay in the settings of predicting peri-operative bleeding in patients on antiplatelet agents who need surgical procedures and in the prediction of treatment response/antiplatelet therapy resistance on the most commonly used antiplatelet drugs. In addition, a limited number of studies suggested that thromboelastometry can be usedfor procedural management of platelet/antipletelet therapy-related bleeding events in the settings of cardiac surgery. Nevertheless, due to limited data and the aforementioned unanswered issues, further research is still needed.

## Figures and Tables

**Table 1:** Thromboelastography (TEG^®^).

**Assay Principle**		Viscoelastic hemostatic assay, uses 340 µL of whole blood in a rotating cylindrical cup while a fixed pin on a torsion wire is suspended in the blood, coagulation is activated by selected activators in a pre-defined assay
**Manufacturer**		Haemoscope, Haemonetics, Niles, IL, USA.
**Platelet Function Testing**		Yes (Platelet mapping assay specifically designed for thromboelastography is commercially available)
**Parameter**	**Calculation**	**Information Provided**
R (Reaction rate)	the time elapsed from the coagulation trigger until the formation of a clot of 2 mm	Time from initiation of clotting process until clot starts to form
K (Kinetics time)	the time elapsed from 2 to 20 mm	Flags the speed of formation of a solid clot
MA (Maximum amplitude)	the maximum amplitude of the signal	Reflects the maximum clot strength
LY30 (Lysis 30)	percentage of remaining clot stability in relation to the MA value at 30 min after R	Reflects loss of clot stability
**Assay**		**Description**
Kaolin		Kaolin acts as contact activators.
Rapid TEG		Reagent contains tissue factor and kaolin as inducers.
HTEG		Reagent with lipophilized heparinase to neutralize unfractionated heparin. Used in conjunction with kaolin to measure heparin effect.
Functional fibrinogen		Reagent with tissue factor and abciximab (glycoprotein IIb/IIIa platelet receptor blocker), inhibiting platelet contribution to clot formation. Allows qualitative measurement of the fibrinogen contribution to clot strength independent of platelets.
Native		Native whole blood sample analyzed following only recalcification. Impractical for clinical use given long R time.
Platelet mapping		Assay uses heparinized blood mixed with ActivatorF (reptilase and activated factor XIII). Sufficient heparin is present to entirely suppress the generation of thrombin while fibrinogen is converted to fibrin and cross- linked due to the presence of reptilase and activated factor XIII. Subsequent addition of either arachadonic acid (AA) or adenosine diphosphate (ADP) allows measurement of the platelet activation response to these inducers in the absence of thrombin. These results are compared to kaolin analysis to determine platelet response to AA and ADP.

**Table 2:** Rotational thromboelastometry (ROTEM^®^).

**Assay Principle**		Viscoelastic hemostatic assay, uses 340 µL of whole blood in a fixed cylindrical cup while a pin suspended on a ball-bearing mechanism oscillates with the application of a constant force, coagulation is activated by selected activators in a pre-defined assays
**Manufacturer**		Instrumentation Laboratory, Bedford, MA, USA
**Platelet Function Testing**		No (FIBTEM indirectly assesses platelet function, platelet mapping assay could be adapted for ROTEM^®^)
**Parameter**	**Calculation**	**Information Provided**
CT (Clotting time)	the time elapsed from the coagulation trigger until the formation of a clot of 2 mm	Time from initiation of clotting process until clot starts to form
CFT (Clot forming time)	the time elapsed from 2 to 20 mm	Flags the speed of formation of a solid clot
MCF (Maximum clot firmness)	the maximum amplitude of the signal	Reflects the maximum clot strength
LI 30 (Lysis index after 30 min)	percentage of remaining clot stability in relation to the MCF value at 30 min after CT	Reflects loss of clot stability
**Assay**	**Activator/Inhibitor**	**Information Provided**
INTEM	Contact activation	Fast assessment of clot forming, fibrin polymerization, and fibrinolysis through the intrinsic pathway
HEPTEM	Contact activation + heparinase	ROTEM measurement without heparin contribution: specific detection of heparin (compared to INTEM), measurement of clotting in heparinized ones
EXTEM	Tissue factor activation	Fast assessment of clot forming, fibrin polymerization, and fibrinolysis through the extrinsic pathway
FIBTEM	Tissue factor activation + platelet inhibition	ROTEM measurement without platelets: qualitative measurement of fibrinogen status
APTEM	Tissue factor activation + aprotinin	In vitro fibrinolysis inhibition: fast detection of lysis when compared with EXTEM
NATEM	Recalcification only = classical TEM (thromboelastometry)	Sensitive measurement of the equilibrium of coagulation activation or inhibition

## Data Availability

All the data are available at Corresponding Author (matej.samos@gmail.com) upon reasonable request.

## Abbreviations

ACS	acute coronary syndromes
ADP	adenosinediphosphate
ADPRB	ADP receptor blockers
ASA	acetylsalicylic acid
CABG	coronary artery bypass graft
CI	confidence interval
GRAVITAS	Gauging Responsiveness with a VerifyNow P2Y12 assay: Impact on Thrombosis and Safety
HPR	high on-treatment platelet reactivity
HR	hazard ratio
LTA	light transmission aggregometry
MPR	maximal platelet reactivity
PCI	percutaneous coronary interventions
PFA	Platelet Function Analyzer
PFT	platelet function testing
POC	point-of-care
PRI	platelet reactivity index
PRP	platelet rich plasma
PRU	platelet response units
ROTEM^®^	thromboelastometry
SCAD	stable coronary artery disease
STEMI	ST elevation myocardial infarction (MI)
TEG^®^	thromboelastography
TRAP	thrombin receptor activating peptide
VASP-P	vasodilator-stimulated phosphoprotein (VASP) phosphorylation
VHA	viscoelastic hemostatic assays
